# Host Response of Winter Wheat to the Causal Agents of Eyespot and Fungicide Resistance of the Pathogens

**DOI:** 10.3390/plants15020285

**Published:** 2026-01-17

**Authors:** Jana Palicová, Pavel Matušinsky, Simona Čejková, Alena Hanzalová, Veronika Dumalasová, Taťána Militká, Dominik Bleša, Jana Chrpová

**Affiliations:** 1 Czech Agrifood Research Center (CARC), Drnovská 507, 161 00 Prague, Czech Republic; alena.hanzalova@carc.cz (A.H.); veronika.dumalasova@carc.cz (V.D.); tatana.militka@carc.cz (T.M.); jana.chrpova@carc.cz (J.C.); 2 Department of Botany, Faculty of Science, Palacký University in Olomouc, Šlechtitelů 27, 783 71 Olomouc, Czech Republic; pavel.matusinsky@upol.cz (P.M.); simona.cejkova@upol.cz (S.Č.); 3 Agrotest Fyto, Ltd., Havlíčkova 2787/121, 767 01 Kroměříž, Czech Republic; blesa@vukrom.cz; 4 Department of Experimental Biology, Faculty of Science, Masaryk University, 625 00 Brno, Czech Republic

**Keywords:** *Oculimacula yallundae*, *Oculimacula acuformis*, *Pch1* gene, molecular markers, qPCR, fungicides, resistance

## Abstract

Eyespot is one of the most important fungal diseases of wheat in the Czech Republic. As part of a long-term study (2015–2024), the occurrence, population structure, and pathogenic variability of *Oculimacula yallundae* and *Oculimacula acuformis* were investigated. In total, 356 *O. yallundae*, 24 *O. acuformis*, and 33 mixed cultures were collected and identified using PCR. The study also included small-plot inoculation trials (2022–2023) to assess the response of widely grown winter wheat cultivars. Disease severity was evaluated visually, pathogen DNA was quantified using qPCR, and the presence of the resistance gene *Pch1* was determined with the STS marker *Xorw1*. In addition to these analyses, monitoring of fungicide resistance to two commonly used fungicides (fluxapyroxad and prothioconazole) was performed. The results showed significant differences among cultivars and seasons. Genotypes carrying *Pch1*—including Annie, Campesino, Illusion, KWS Donovan, LG Absalon, and Pallas—exhibited the lowest levels of infection, whereas Mercedes and Dagmar were the most susceptible. The qPCR reliably detected and distinguished both pathogens, with *O. yallundae* occurring at higher concentrations. Fungicide sensitivity testing revealed EC_50_ values (mean ± SD) of 0.09 ± 0.13 μg·mL^−1^ for fluxapyroxad and 0.30 ± 0.22 μg·mL^−1^ for prothioconazole, indicating that eyespot pathogens remain largely sensitive, with only minor signs of reduced sensitivity.

## 1. Introduction

The issue of cereal stem base diseases remains relevant due to the widespread cultivation of cereals, the effects of global warming, the EU’s trend toward restricting the use of fungicides, and the emergence of fungicide resistance. Eyespot, caused by the ascomycete fungi *Oculimacula yallundae* and *Oculimacula acuformis,* is a persistent threat to winter wheat production in temperate regions. These pathogens induce lesions on stem bases that can lead to significant yield losses, particularly in environments conducive to disease development. There are differences relating to host pathogenicity, with *O. yallundae* being more pathogenic on wheat than on barley and only causing slight disease symptoms on rye, whereas *O. acuformis* is almost equally pathogenic on all these species [1]. Surveys in several European regions often report a predominance of *O. yallundae* in wheat, with frequent co-occurrence of *O. acuformis* [2,3,4]. However, the ratio of the two species varies regionally and annually, depending on agronomic factors, previous crops, cultivated varieties, and fungicide management. Due to the different sensitivity to fungicides and different epidemiological characteristics of both species, the cooccurrence is important for protection management decisions.

Accurate diagnosis of eyespot can be challenging due to the overlap of symptoms with those of other stem-base diseases and the coexistence of both *Oculimacula* species. Traditional visual assessments are insufficient for reliable identification, as the pathogens differ in pathogenicity and fungicide sensitivity. Recent advances in molecular diagnostics, such as quantitative PCR (qPCR) and loop-mediated isothermal amplification (LAMP), have enabled the rapid and precise detection of *O. yallundae* and *O. acuformis* in plant tissues and environmental samples, thereby greatly improving pathogen monitoring and supporting resistance breeding programs [5,6]. However, the effective application of these molecular tools requires their integration with classical phenotypic assessments, including visual disease evaluation and the detection of specific resistance-related genes. A comprehensive approach combining modern molecular technologies with traditional phenotyping provides a more robust and biologically relevant framework for understanding host–pathogen interactions and assessing resistance under practical conditions.

Management of eyespot has relied heavily on fungicide applications, particularly systemic active ingredients such as triazoles and imidazoles. However, the widespread use of these chemicals since the 1970s has led to the selection of resistant strains of *Oculimacula* spp. Notably, resistance to benzimidazole fungicides prompted a shift to sterol 14α-demethylation inhibitors (DMIs), but resistance to these, including prochloraz and triazoles, has also been documented. In the Czech Republic, a medium to high level of *Oculimacula* spp. resistance to prochloraz was observed during monitoring from 2012 to 2017 [7,8].

The DMI class provides systemic activity needed to penetrate stem bases and has been highly effective against eyespot when applied at the recommended GS30–32 timing [2,9]. An anilinopyrimidine fungicide (cyprodinil) was also introduced in the 1990s to manage DMI-resistant eyespot in some regions [2]. More recently, succinate dehydrogenase inhibitor (SDHI) fungicides have been deployed for eyespot control, notably boscalid and newer actives such as fluxapyroxad, often in co-formulations with DMIs to broaden their efficacy [10]. Field and laboratory efficacy tests demonstrate that DMI–SDHI mixtures can achieve high levels of eyespot suppression. For example, a triple combination of epoxiconazole (DMI) + fluxapyroxad (SDHI) + pyraclostrobin (QoI) provided the greatest mycelial growth inhibition in *Oculimacula* isolates, whereas a DMI–QoI mixture (prothioconazole + trifloxystrobin) was significantly less effective [10]. Azole (DMI) fungicides—especially prothioconazole—remain the most consistently effective solo actives for eyespot, and SDHI co-applications further enhance control, provided that applications target the critical stem base infection window at GS 31–32 [2,9].

Although fungicides can effectively manage eyespot, pathogen populations may present resistance under selective pressure. The eyespot fungi have a history of developing resistance to single-site pesticides. Practical resistance to benzimidazoles became prevalent by the mid-1980s, and subsequently, *O. acuformis* and *O. yallundae* populations with reduced DMI sensitivity were detected in the 1990s and 2000s [2]. Molecular studies have revealed that azole resistance in *Oculimacula* is polygenic, often involving cumulative mutations in the fungal *CYP51* sterol demethylase target, which confer quantitative decreases in DMI sensitivity [11]. Common *CYP51* amino acid alterations (e.g., V136A, I381V) associated with moderate azole resistance have been documented in field isolates, leading to a gradual shift in in vitro sensitivity without complete loss of field control [11]. Despite these trends, current DMI fungicides continue to perform well in the field—for instance, prothioconazole has largely retained its efficacy across Europe, likely due to its unique binding mechanism and the continued low frequency of high-resistance alleles in *Oculimacula* populations [12]. Similarly, eyespot management with SDHI fungicides remains effective to date, with no significant SDHI resistance reported; baseline sensitivity surveys up to 2019 indicate that *Oculimacula* isolates are uniformly sensitive to SDHIs [12]. Nevertheless, both DMIs and SDHIs are classified as medium- to high-risk for resistance development because they act on specific pathogen targets [12]. Therefore, resistance management strategies are essential, including mixing DMIs with SDHIs or multi-site partners, limiting the number of applications, and avoiding reduced-dose treatments that could allow survival of less sensitive strains [12,13]. This highlights the need to develop integrated strategies for protecting winter wheat crops and to breed varieties resistant to eyespot.

At the molecular level, resistance to eyespot in wheat is primarily governed by several major genes, *Pch1* through *Pch4*, each with distinct origins and mechanisms. The *Pch1* gene, introgressed from *Aegilops ventricosa*, is the most effective and widely used resistance gene and is routinely detected in breeding programs using the molecular marker *Xorw1* [14]. *Pch2*, derived from the wheat cultivar Cappelle Desprez [15], and *Pch3*, from *Triticum monococcum*, provide additional sources of resistance [16]. Most recently, the *Pch4* gene was discovered in *Aegilops tauschii*, thereby expanding the genetic toolkit for breeding [17]. The quantitative trait locus (QTL) *Q.Pch.jic-5A* has been identified as an important genetic factor contributing to eyespot resistance in wheat [18]. However, most winter wheat varieties cultivated in Central Europe still lack these resistance genes. This underscores the importance of ongoing breeding efforts and molecular monitoring.

This study focused on the eyespot causal agents and host response in winter wheat cultivars, including the role of the resistance gene *Pch1* and its frequency in cultivars grown in the Czech Republic and other European countries (France, Germany, Great Britain, Italy, Hungary, Slovakia, etc.). The quantitative real-time PCR (qPCR) was used to verify the visual assessment. Moreover, the susceptibility of the pathogens to selected fungicides was tested. The other part of the present study was undertaken to evaluate the efficacy of current fungicide options—particularly representative DMI and SDHI actives—for eyespot control in cereals, and to assess the sensitivity of contemporary *Oculimacula* populations through laboratory EC_50_ assays. By linking fungicide performance with pathogen sensitivity data, the study aims to inform resistance management strategies for sustainable control of eyespot disease.

## 2. Results

### 2.1. Occurrence of Oculimacula *spp.*

**Figure 1:**
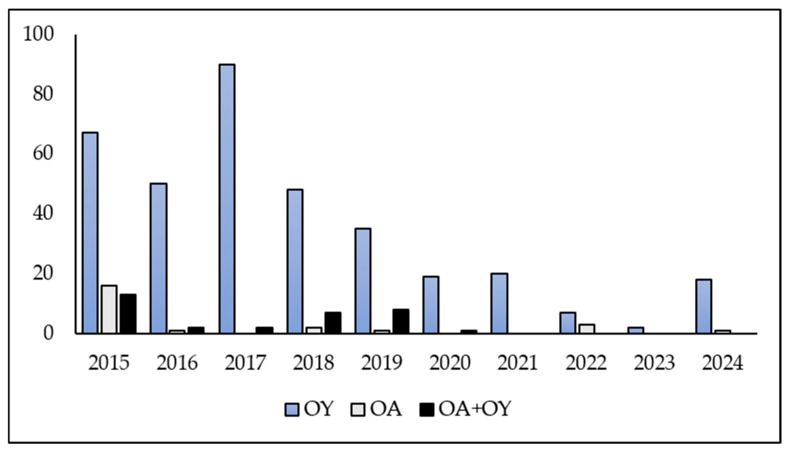
The *Oculimacula* spp. isolates originated from the Czech Republic in 2015–2024 (OY—*O. yallundae*, OA—*O. acuformis*, OA + OY—mixed cultures of both species).

Ten years of data clearly show that eyespot represents one of the most significant fungal diseases affecting the stem bases of wheat in the Czech Republic. A total of 413 isolates of *Oculimacula* spp. were obtained from winter wheat samples infected with eyespot during the period 2015–2024 (Figure 1). The samples were collected from the most important wheat-growing regions in the Czech Republic. The species of *Oculimacula* were identified by PCR. A predominance of *O. yallundae* isolates (356) was observed compared to *O. acuformis* isolates (24) and mixed cultures containing both species (33). During the 10-year period, *O. acuformis* was found to predominate on wheat in only one location. This location was an agricultural area in the southwestern part of the Czech Republic that was severely affected by eyespot in 2015 due to the ineffectiveness of the applied fungicide based on a combination of two active substances, prochloraz and propiconazole.

### 2.2. Cultivar Reaction to Eyespot

#### 2.2.1. Visual Assessment of Eyespot Symptoms

Statistically significant differences were observed in the response of the tested winter wheat cultivars to inoculation with *Oculimacula* spp. during the years 2022–2023 (Kruskal–Wallis test, *H* = 25, *p* < 0.001). Cultivars carrying the eyespot resistance gene *Pch1* generally exhibited fewer disease symptoms compared to those lacking *Pch1*. In resistant cultivars with *Pch1*, the symptoms manifested as nonspecific, necrotic spots at the stem base, which differed from the typical, elliptical, eye-shaped lesions. The cultivars Annie (resistant control), Illusion, Pallas, KWS Donovan and Campesino showed the lowest levels of disease symptoms among all tested cultivars in both years. Of the cultivars possessing the *Pch1* gene, cultivar LG Absalon was the most attacked by eyespot. In 2023, the symptoms on LG Absalon were more severe than those on the Fakir cultivar, which lacks the *Pch1* gene. Among the cultivars lacking the *Pch1* gene, Adina, Fakir, and LG Dita were the least affected in both years, followed by Nonstop, LG Mocca, Genius, Butterfly, and so on (Table 1).

In some cultivars, the incidence of eyespot infestation varied significantly from year to year. In some cases, the level of infestation varied by more than 0.5 on the 0–5 scale (Illusion, Asory, LG Mondial, Kalbex, Crossway, Skif, and Dagmar). The most intensive eyespot symptoms were observed in cultivars Mercedes, Dagmar, Julie, RGT Sacramento, and Skif.

Of the 26 winter wheat cultivars evaluated, 8 cultivars can be considered resistant to eyespot according to the 0–5 scale used (0–2 resistant, 3 moderately resistant to moderately susceptible, 4–5 susceptible). Six of them carry the *Pch1* resistance gene, and two cultivars, Adina and Fakir, do not. Other cultivars had an average infection value of 3 over two years, showing a moderately resistant to moderately susceptible reaction. However, the cultivars Asory, Kalbex, Crossway, Skif, Dagmar, and Mercedes showed a susceptible reaction in one year.

#### 2.2.2. qPCR Quantification of *Oculimacula* spp.

**Figure 2:**
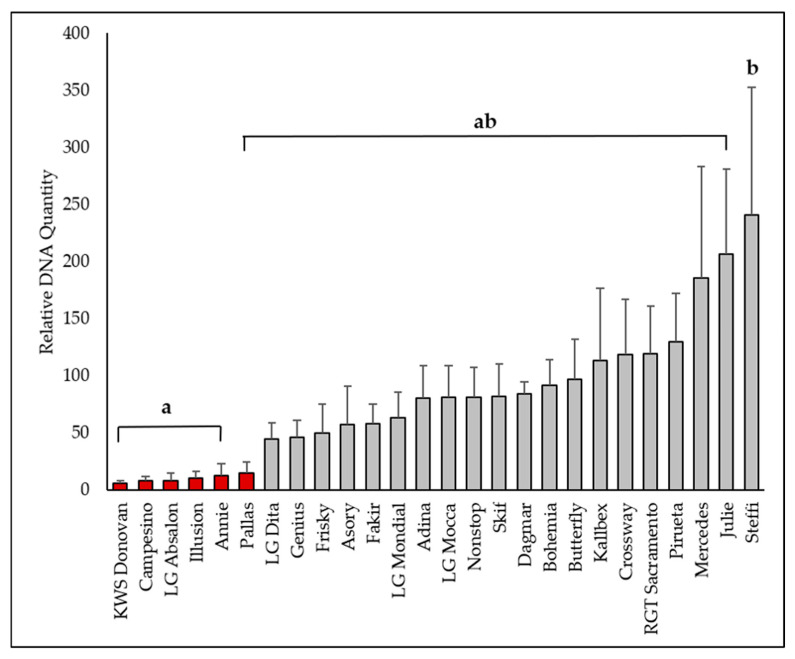
The qPCR assessment of *Oculimacula yallundae* in the winter wheat cultivars (2022–2023). Columns represent means, bars SD. In the ANOVA with a multiple comparison Tukey’s HSD test (*p* < 0.05) on Box-Cox transformed data (*F* = 3.478; *p* < 0.001), homogeneous groups are marked with the same letters. Red columns indicate cultivars carrying the *Pch1* resistance gene.

Using quantitative polymerase chain reaction (qPCR) analysis, significant differences in *O. yallundae* were revealed among the tested cultivars (samples) during the 2022–2023 period (Figure 2). The cultivar Annie (carrying the eyespot resistance gene *Pch1*) served as the control, and the relative amount of *O. yallundae* DNA in other cultivars was expressed as a fold difference (FD) compared to this control. The lowest DNA levels were found in KWS Donovan (*Pch1*, FD 5.84), as well as in Campesino (*Pch1*, FD 8.15), LG Absalon (*Pch1*, FD 8.61), Illusion (*Pch1*, FD 10.77), Annie (*Pch1*, FD 12.90), and Pallas (*Pch1*, FD 14.87). The highest DNA concentrations were detected in Steffi (FD 240.65), Julie (FD 206.40), and Mercedes (FD 185.59).

**Figure 3:**
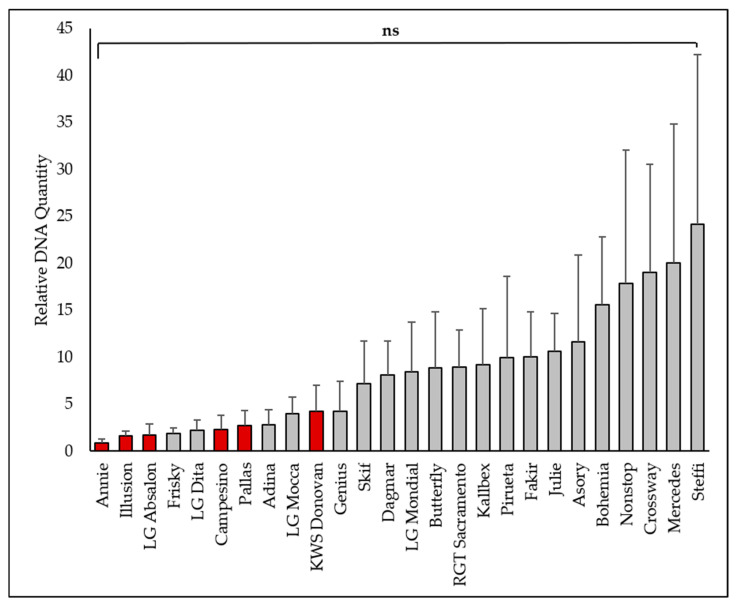
The qPCR assessment of *Oculimacula acuformis* in the winter wheat cultivars (2022–2023). Columns represent means, bars represent SD. The ANOVA (*F* = 1.098; *p* = 0.366) was not statistically significant (ns). Red columns indicate cultivars carrying the *Pch1* resistance gene.

*Oculimacula acuformis* was detected at much lower levels than *O. yallundae*, and differences among cultivars were not statistically significant (Figure 3). The lowest amounts of *O. acuformis* DNA were observed in Annie (*Pch1*, FD 0.91), Illusion (*Pch1*, FD 1.62), LG Absalon (*Pch1*, FD 1.70), and Frisky (FD 1.85). The highest levels were found in Steffi (FD 24.18), Mercedes (FD 20.08), and Crossway (FD 19.04). According to the qPCR assessment, cultivar Steffi was the most infected cultivar for both *O. yallundae* and *O. acuformis*.

**Figure 4:**
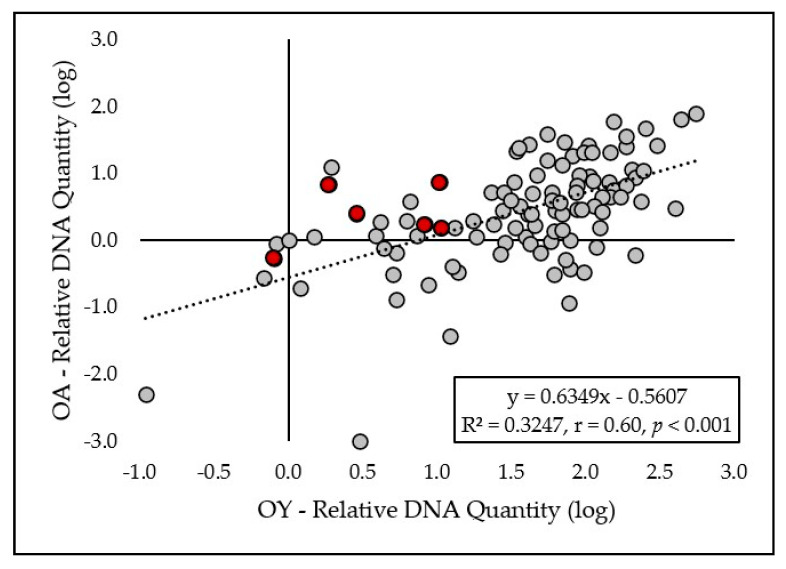
The relationship between the log-transformed relative quantity of *O. acuformis* DNA and *O. yallundae* DNA obtained by qPCR. Red dots indicate cultivars carrying the *Pch1* resistance gene.

A moderately high linear correlation (Figure 4) between relative DNA quantity of both eyespot pathogens in plant tissues was observed in 2022–2023 (R^2^ = 0.3247, r = 0.60, *p* < 0.001).

#### 2.2.3. Correlation Between Visual Assessment and qPCR

**Figure 5:**
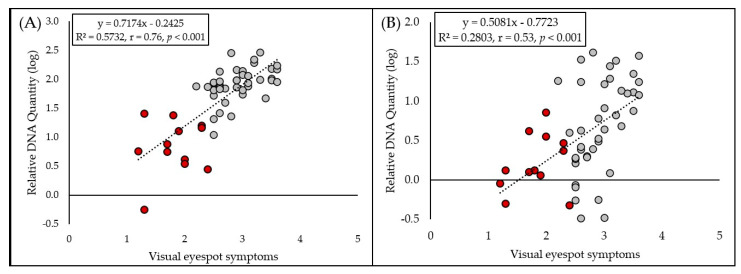
Relationship between visual eyespot symptom scores and the log-transformed relative DNA quantity of (**A**) *Oculimacula yallundae* and (**B**) *O. acuformis*, as determined by qPCR. Red dots indicate cultivars carrying the *Pch1* resistance gene.

A high linear correlation (Figure 5A) between visual eyespot symptoms and pathogen *O. yallundae* DNA content in plant tissues was observed in 2022–2023 (R^2^ = 0.5732, r = 0.76, *p* < 0.001). In the case of *O. acuformis*, only moderate linear correlation (Figure 5B) was observed (R^2^ = 0.2803, r = 0.53, *p* < 0.001).

### 2.3. Resistance of Oculimacula Isolates to Fungicides

**Figure 6:**
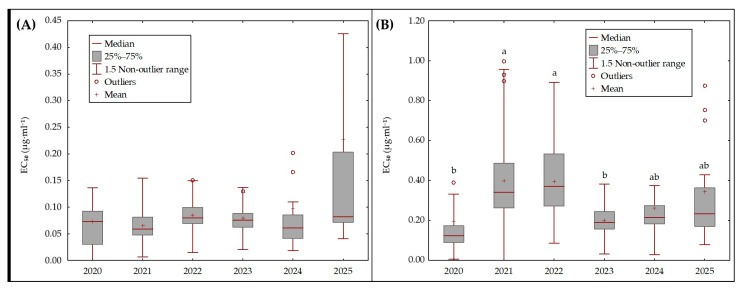
Boxplots of in vitro EC_50_ of *Oculimacula* spp. isolates for fluxapyroxad (**A**) and prothioconazole (**B**). Each box indicates the interquartile range (25th–75th percentile), the horizontal line represents the median, and the cross marks the mean. Whiskers represent the non-outlier range, approximately corresponding to 1.5 × IQR (Tukey’s definition). Outliers are plotted as individual points. Different letters above the boxes indicate statistically significant differences between years, as determined by multiple pairwise comparisons; for fluxapyroxad, no significant pairwise differences were detected.

Isolates of *Oculimacula* spp. exhibited low levels of reduced sensitivity. The highest proportion of isolates with elevated EC_50_ values occurred in years with increased disease pressure, although the absolute ED_50_ estimates for *Oculimacula* spp. remained consistent across all survey years. For fluxapyroxad, EC_50_ values differed significantly across years (Kruskal–Wallis *H* = 14.18; *p* = 0.0145). Despite this overall effect, subsequent pairwise comparisons did not reveal any statistically significant differences between individual years (Figure 6A). Thus, although some inter-annual variability was observed, it did not follow a consistent directional pattern and likely reflects background variation within field populations rather than a systematic shift in sensitivity. In contrast, a clearer temporal pattern was observed for prothioconazole. EC_50_ values differed significantly among years (Kruskal–Wallis *H* = 51.72; *p* < 0.001), and multiple pairwise comparisons identified several year groups with distinct sensitivity profiles (Figure 6B). Isolates collected in 2021 and 2022 displayed significantly higher EC_50_ values than isolates from 2020 and 2023 (*p* < 0.05). In later years (2024–2025), median EC_50_ values stabilized at intermediate levels, suggesting population sensitivity. The observed variability appears to reflect sampling limitations rather than a consistent or significant shift in response to prothioconazole.

## 3. Discussion

### 3.1. Occurrence of Eyespot Pathogens

*O. yallundae* was found to be the dominant species responsible for eyespot in nearly all surveyed regions of the Czech Republic, with only one exception (see below). Occasionally, both species of *Oculimacula* were found in the same wheat stem. Research in Lithuania also confirmed the predominance of *O. yallundae* in wheat, with both species sometimes co-occurring; however, *O. acuformis* was less frequent [19]. The French study analyzed populations of eyespot fungi and found that *O. yallundae* was generally more prevalent than *O. acuformis* in wheat fields [2]. The authors also discussed how fungicide use and agronomic practices can influence the species ratio. In the United Kingdom, Bierman et al. [20] observed a consistent balance of approximately 50% of each species in both untreated and carbendazim-treated plots over five consecutive seasons.

The predominance of *O. yallundae* in fields with a high incidence of eyepot in the Czech Republic is, to some extent, positive, as *O. acuformis* is more often associated with the development of resistance to fungicides [2,21]. Fungicide resistance in eyespot is closely linked to population structure, as confirmed by Leroux et al. [2]. The predominance of *O. acuformis* was detected only in one field in the southwestern part of the Czech Republic in 2015, which resulted in low efficacy of protection with a fungicide based on a combination of two active substances, prochloraz and propiconazole, even though it was applied at the correct time and in the recommended dose. In long-term studies in Western Europe, reduced sensitivity to prochloraz was more common in *O. acuformis*, resulting in a higher proportion of this species in populations exposed to repeated fungicide applications [2,21,22].

### 3.2. Cultivar Resistance

The results clearly demonstrate that the presence of the *Pch1* gene significantly reduces eyespot severity in winter wheat cultivars, confirming its importance in resistance breeding programs. The previous studies have repeatedly shown that the *Pch1* gene confers effective resistance against eyespot, often resulting in atypical symptom expression such as nonspecific necrotic spots rather than the typical elliptical lesions [23,24,25]. However, variability among *Pch1* cultivars indicates that genetic background and environmental factors can affect the effectiveness of this resistance. Interestingly, some *Pch1*-lacking cultivars exhibited low disease levels, suggesting the presence of additional, potentially polygenic, resistance mechanisms. These results highlight the importance of diversifying resistance sources and integrating major genes and quantitative traits into breeding programs. Furthermore, year-to-year fluctuations in disease incidence highlight the strong influence of environmental conditions, emphasizing the need for ongoing monitoring and adaptive management strategies.

Interestingly, while most *Pch1* cultivars showed high resistance, some variation was observed within this group. For example, LG Absalon, despite carrying *Pch1*, was more infected (visual symptoms) than other *Pch1* cultivars, and in 2023 even surpassed the susceptible cultivar Fakir in symptom severity. This suggests that the expression of *Pch1*-mediated resistance may be influenced by genetic background or environmental factors, as also noted by Koen et al. [24] and Fountaine et al. [26].

In this context, it should be noted that inoculation was performed using a mixed-isolate inoculum, which was intended to increase the likelihood of successful infection and ensure phenotypic expression across diverse genotypes under field conditions. However, this approach limits the ability to assign causality to specific pathogen strains. The atypical reaction of LG Absalon may reflect either host genetic background effects or a differential response to specific virulence traits. Among cultivars lacking *Pch1*, Adina and Fakir showed unexpectedly low levels of disease, indicating the presence of additional, possibly quantitative, resistance factors. Such findings align with reports that some cultivars without *Pch1* can exhibit moderate resistance, likely due to polygenic effects [27].

Despite consistent conditions in the small plot experiments, infection levels fluctuated from year to year. The observed variation in eyespot incidence among certain cultivars (e.g., Asory, Crossway, Illusion, Kalbex, Skif) highlights the strong influence of environmental conditions on disease development. This variability is well-documented in literature, where factors such as temperature, humidity, and inoculum pressure can significantly affect both symptom expression and pathogen multiplication [1,28]. Illusion was the only cultivar with the gene *Pch1* with a variable reaction to eyespot inoculation. Therefore, other resistant genes, *Pch2*, *Pch3*, *Pch4*, and QTLs, also play a significant role in the overall expression of the cultivar. Recent studies on *Pch4* offer a new type of eyespot resistance, which differs from that of *Pch1*/*Pch2*. It can be utilized in a resistance pyramiding strategy, which combines multiple genes and QTLs for stronger and more stable protection [17]. Further research is necessary to identify other sources of resistance in the genotypes of cultivated varieties, particularly the previously described *Pch2*–*Pch4* genes and QTLs.

The qPCR results revealed that *O. yallundae* was detected at much higher levels than *O. acuformis* across all cultivars, and significant differences among cultivars were found only for *O. yallundae*. *O. acuformis* probably played a minimal role in infection, even though the inoculum levels of both fungi were similar. The strongest infections (with the highest DNA content) were observed in cultivars Steffi, Julie, and Mercedes, which also exhibited the most severe symptoms. The correlation between qPCR data and symptom severity is supported by the high linear regression found for *O. yallundae* (r = 0.76), in line with previous findings that molecular quantification can reliably reflect field resistance [3,26].

The high linear correlation between visual symptoms and *O. yallundae* DNA content indicates that visual scoring remains a useful tool for resistance screening, though it may underestimate or overestimate infection in some cases. The weaker correlation for *O. acuformis* likely reflects its low abundance and the limited variation among cultivars. These findings are consistent with studies suggesting that molecular methods provide a more sensitive and objective measure of pathogen presence, especially for less present species [26].

Overall, these results confirm the effectiveness of the *Pch1* gene in conferring resistance to eyespot, particularly against *O. yallundae*, and highlight the importance of integrating both visual and molecular assessments in resistance breeding. The predominance of *O. yallundae* and the low incidence of *O. acuformis* suggest that current breeding and management strategies should focus primarily on the former. However, ongoing monitoring is warranted, as shifts in species composition can occur in response to fungicide use and environmental change [2,19].

### 3.3. Resistance of Eyespot Causal Agents to Fungicides

The EC_50_ assay results indicate that eyespot pathogens in winter wheat remain largely sensitive to the tested fungicides, with only minor evidence of reduced sensitivity emerging over the study period. All field isolates of *Oculimacula* spp. exhibited low levels of EC_50_ elevation relative to baseline. For fluxapyroxad (SDHI fungicide), EC_50_ distributions varied significantly across years, but no individual year differed significantly in post-hoc comparisons. This implies only subtle, inconsistent shifts in fluxapyroxad sensitivity over time. In contrast, prothioconazole (a DMI/azole) exhibited a pronounced but transient increase in EC_50_ from 2021 to 2022. Isolates from those two years had significantly higher EC_50_ values than those from 2020 and 2023. By 2024–2025, EC_50_ values had stabilized to intermediate levels. This pattern suggests that disease pressure in 2021–2022 may have selected isolates with slightly reduced prothioconazole sensitivity. It should be noted that the small sample sizes in certain years may have limited the statistical power to detect subtle differences, so these year-to-year trends should be interpreted with caution. To enhance the interpretability of resistance monitoring data, we propose a provisional EC_50_ threshold of 0.5 µg·mL^−1^ for prothioconazole. This value reflects the upper bound of baseline sensitivity observed in our dataset and aligns with other European studies using similar methodologies [2,10,22]. Exceeding this threshold could serve as an early warning indicator of emerging reduced sensitivity in *Oculimacula* populations. Although none of the yearly median EC_50_ values surpassed this level, several individual isolates from 2021 to 2022 approached or slightly exceeded it, reinforcing the need for continued vigilance.

These findings are broadly consistent with the literature on fungicide resistance dynamics in eyespot. Long-term surveys in Europe have documented cases of fungicide resistance in *Oculimacula* populations, primarily to older chemicals or following prolonged use. For example, benzimidazole fungicides such as carbendazim had already been selected for widespread resistance in eyespot populations by the 1990s. Similarly, resistance to the imidazole DMI fungicide prochloraz became well established in both *O. acuformis* and *O. yallundae* across many regions [2]. This was often accompanied by shifts in species composition—prochloraz use tends to select for the more tolerant *O. acuformis* over *O. yallundae* [19]. In this study, by contrast, prothioconazole remained effective, which aligns with reports that no field resistance to prothioconazole had yet been detected in eyespot populations [2]. Prothioconazole is a triazolinethione DMI with a slightly different binding mode to the fungal 14α-demethylase (*CYP51*) enzyme, thought to allow control of some DMI-resistant strains that harbor typical azole-target mutations [2,11]. An interesting phenomenon was observed in France, where a few *O. yallundae* isolates with multidrug resistance (MDR)—exhibiting slightly decreased sensitivity to both azoles and SDHIs—were identified in the 2000s, albeit at low frequencies [2]. Likewise, Czech researchers found that while a majority of eyespot isolates had low to moderate resistance to prochloraz by the late 2010s, the same isolates remained fully sensitive to prothioconazole and SDHIs [10]. Taken together, the data presented here and these studies indicate that fluxapyroxad and prothioconazole continue to provide robust eyespot control, with only incremental shifts in sensitivity so far. This underscores the importance of ongoing monitoring and strategic management to prevent further escalation.

Maintaining the long-term efficacy of these fungicides will require diligent resistance management, especially given the lessons learned from other pathogens and active ingredients. Fungicide resistance is an evolutionary response to selection pressure [29], and even “moderate risk” compounds, such as azoles, can eventually select for less sensitive subpopulations if overused or applied insufficiently. The azoles have, in fact, provided relatively durable disease control for decades [30], but instances of partial resistance (e.g., azole-resistant *Zymoseptoria* and *Oculimacula* with *CYP51* mutations) illustrate that their longevity should not be taken for granted [2,11]. Best practices to slow selection of resistant strains include using fungicides in mixtures and rotation, limiting the number of applications, and applying at recommended full doses at optimal timing [31,32]. In eyespot management, mixing prothioconazole with a complementary mode of action such as fluxapyroxad is advisable. Combination treatments exploit the lack of cross-resistance between azoles and SDHIs, making it less likely for the pathogen to simultaneously develop tolerance to both [11,31]. Indeed, field studies have demonstrated that such mixtures can achieve high efficacy and delay the development of resistance. For instance, a three-way azole + SDHI + QoI mix provided the best control in *Oculimacula* trials, whereas prolonged use of azole + QoI (trifloxystrobin) resulted in a drop in efficacy once QoI-resistant strains emerged [10]. It is also important to continue regular sensitivity monitoring (e.g., annual EC_50_ testing of isolates) to observe any shifts early and adjust recommendations accordingly [2,10]. Finally, an integrated disease management approach is crucial, as using eyespot-resistant wheat varieties (genes *Pch1* and *Pch2*), crop rotation, and ensuring fungicides are only applied, when necessary, can all reduce the selective pressure on the pathogens [29,33]. Notably, Ray et al. [33] observed that slight to moderate eyespot infections caused minimal yield loss, implying that routine fungicide sprays in low-disease situations may be unwarranted. Avoiding such unnecessary applications helps preserve the effectiveness of fungicides. The findings presented here confirm that current fungicide options still perform well against eyespot. By heeding the resistance management principles drawn from decades of research, eyespot can be managed effectively and sustainably in winter wheat.

## 4. Materials and Methods

### 4.1. Sampling of Stem Bases and Fungal Isolates

Samples of winter wheat stems infected with eyespot were collected in various parts of the Czech Republic from March to June in 2015–2024. The samples came from farms, small growers, breeding stations, and the Central Institute for Supervising and Testing in Agriculture. Some samples were collected from fields with a high incidence of eyespot, despite proper fungicide application. Typical symptoms used to identify eyespot were eye-shaped lesions (Supplementary Figure S1) on stem bases, usually located just above the soil surface. The center of the lesions was brown to black. Stem bases were cut from the infected wheat plants, surface-sterilized in a 5% NaOCl for 2 min and rinsed three times in sterile distilled water [3]. Subsequently, the stem segments were placed on commercial potato dextrose agar (PDA-Himedia, Mumbai, India) containing antibiotics to inhibit bacterial growth (ampicillin 50 μg·mL^−1^ or chloramphenicol 100 µg·mL^−1^). In the case of gray mycelium inside the stem, which is typical of eyespot, the fungal pathogen was transferred directly to PDA with antibiotics. This method of isolation was the most effective. Petri dishes were incubated at 18 °C under UV-C light for 5 days. The developing colonies were transferred to new PDA plates and the *Oculimacula* isolates obtained were stored in the CARC (Czech Agrifood Research Center) working culture collection of the Genetics and Breeding Methods team for future testing. Selected isolates are gradually added to the CARC collection of microorganism cultures, Acronym: VURV, in Prague.

### 4.2. Determination of Oculimacula *spp.* By PCR

*O. yallundae* and *O. acuformis* differ in morphology, pathogenicity, occurrence, and sensitivity to fungicides [34]. Although colony color is highly variable in both species and therefore not a reliable discriminative trait, marked differences were observed in colony margin morphology. While *O. yallundae* typically formed colonies with smooth and regular margins, *O. acuformis* exhibited irregular, feathery colony edges, as consistently observed in our isolates (Supplementary Figure S2). *Oculimacula* spp. isolates were analyzed using molecular diagnostic methods to distinguish between *O. yallundae* and *O. acuformis*, as morphological characteristics alone often do not provide sufficient resolution for reliable identification. Fungal mycelia from 14-day cultures were disrupted in liquid nitrogen to obtain a homogeneous fine powder, and total genomic DNA was then extracted using the DNeasy^®^ Plant Mini Kit (Qiagen, Venlo, The Netherlands) according to the manufacturer’s protocol and the concentration of DNA was measured by Qubit fluorometer (ThermoFisher Scientific, Waltham, MA, USA). Species-specific primer sets targeting diagnostic *loci* for *O. yallundae* and *O. acuformis*, previously validated by Walsh et al. [6]. Each PCR assay included DNA extracted from infected stem base tissue, a negative control without template, and positive controls consisting of purified genomic DNA from reference cultures of both *Oculimacula* species. Amplification products (10 µL per sample) were separated on 1.7% agarose gels prepared in TAE buffer and visualized after staining with ethidium bromide.

### 4.3. Cultivar Resistance to Eyespot

The reaction of the 26 selected winter wheat cultivars (Table 1) to inoculation with *O. yallundae* and *O. acuformis* was studied in a small plot trial in Prague-Ruzyně (50.0864797 N, 14.3020897 E) in two farming seasons: 2021/2022 and 2022/2023. All tested cultivars were registered in the Czech Republic by the Central Institute for Supervising and Testing in Agriculture. They were also screened with the STS marker *Xorw1* [35] to identify the presence or absence of the *Pch1* gene, as described by Dumalasová et al. [36]. The resistant control was cultivar Annie, which possesses the *Pch1* gene. Each cultivar was sown in two rows (1.5 m) during the autumn sowing period (September/October), with two replications.

The inoculum for the small plot trial was prepared using a mixture of 15 isolates of *O. yallundae* and 7 isolates of *O. acuformis*, as described by Palicová et al. [3]. All the *Oculimacula* isolates used were obtained from various locations in the Czech Republic. Fungi were cultivated on PDA (Himedia) at 20 °C in the dark for 14 days. Mycelium with agar was cut into 5 × 5 mm squares and each Erlenmeyer flask containing sterilized barley grains was inoculated with 4 squares of one *Oculimacula* isolate. The barley grains inoculated with *Oculimacula* spp. were incubated under UV light at 18 °C for about 4–5 weeks. After this time, the inoculum was removed from the flasks, mixed in a large container and directly applied on experimental plots in December and in March (40 g·m^−2^). The reaction of tested cultivars was rated at the milk growth stage (BBCH 73–77). A 0 to 5 rating scale was used (0—no symptoms; 5—broken stem). In inoculated plots, 60 randomly selected stems in two replications were assessed. Quantitative real-time PCR (qPCR) was performed according to the methodology described by Palicová et al. [3]. Stem base fragments (5 cm in length) were ground to a fine powder and homogenized. A total of 200 mg of the resulting powder was used for DNA isolation. DNA samples were analyzed using the universal reverse primer Oculimacula-R (5′-ATT CAA GGG TGG AGG TCT GRA C-3′) together with species-specific primers Ac F-D (5′-GCC ACC CTA CTT CGG TAA-3′) and Yall F-H (5′-GGG GGC TAC CCT ACT TGG CAG-3′) Walsh et al. [6], while the *phenylalanine ammonia-lyase* gene served as the reference. The specificity of the primers was verified by melting analysis. The data were analyzed using the 2^−ΔΔCq^ method with CFX Manager 3.0 software (Bio-Rad, Hercules, CA, USA).

### 4.4. Reaction of Oculimacula Isolates to Fungicides

Fungal isolates used in this study originated from a long-term survey conducted in the Czech Republic from 2020 to 2025, assessing fungicide sensitivity in stem base pathogens of winter wheat. Each year, sampling was conducted at growth stage BBCH 31–32 (typically mid-April) in commercial fields treated according to standard farm fungicide regimes. From every selected field, a single composite sample was collected by harvesting 50–100 plants. Within each field, a 15 × 15 m plot was delineated, and plants were sampled at randomly chosen points along two approximately parallel zigzag transects. Stems exhibiting pronounced basal lesions were retained for pathogen isolation and identification. Basal stem sections (1.5 cm) were excised, surface-sterilized in 5% NaOCl for 2 min, and rinsed three times in sterile distilled water. Segments were placed on potato dextrose agar (PDA) amended with ampicillin (50 μg·mL^−1^) to suppress bacterial growth. Plates with basal stems were incubated in darkness for 3 days, followed by 4 days under near-UV illumination, with 12 h light–dark cycles at a constant temperature of 15 °C. Emerging colonies were identified to the genus or species level (*Oculimacula*, *Microdochium*, or others) based on differential macromorphological traits. Axenic cultures of *Oculimacula* spp. were subcultured onto fresh PDA containing ampicillin and maintained for subsequent fungicide sensitivity assays. Each individual *Oculimacula* spp. isolate obtained from a composite field sample was considered an independent biological sample. In individual years, 2020 (*n* = 30), 2021 (*n* = 39), 2022 (*n* = 22), 2023 (*n* = 24), 2024 (*n* = 17), and 2025 (*n* = 13) isolates were obtained. The inhibitory effects of fluxapyroxad and prothioconazole on mycelial growth of *Oculimacula* spp. were quantified using an agar dilution assay. Isolates were pre-cultured on PDA at 20 °C for 10 days in darkness. Mycelial plugs (1.5 mm diameter) excised from actively growing colony margins were transferred onto PDA plates supplemented with one of five fungicide concentrations (0.0, 0.01, 0.1, 1.0, and 10.0 μg∙ml^−1^). Each isolate × concentration combination was replicated four times. Plates were incubated at 18 °C for 14 days, after which colony diameters were measured. Dose–response relationships were analyzed by probit regression to estimate ED_50_ values following Finney [37].

### 4.5. Statistical Analysis

Statistical analyses were performed in STATISTICA 14 (TIBCO Software Inc., Palo Alto, CA, USA). A non-parametric Kruskal–Wallis test was used to analyze the cultivar data on visual eyespot symptoms. The data of qPCR were analyzed by one-way ANOVA and Tukey’s Honestly Significant Difference (HSD) test for multiple comparisons (*p* < 0.05). Prior to ANOVA, the normality of the residuals was tested using the Shapiro–Wilk test and the homogeneity of variances was tested by combined Bartlett’s, Cochran’s, and Hartley’s tests. The relationship between visual eyespot symptoms and the DNA content of pathogens in plant tissue was described using regression analysis, and logarithmic data transformation was used. Given the distributional characteristics and variance heterogeneity of EC_50_ values, the data were subjected to a non-parametric Kruskal–Wallis test followed by multiple pairwise comparisons_0.05_ to test differences of isolates to fungicides among years.

## 5. Conclusions

In summary, long-term monitoring, combined with molecular diagnostics, has shown that eyespot, predominantly caused by *O. yallundae* in wheat, remains one of the most significant fungal diseases affecting winter wheat in the Czech Republic. The presence of the resistance gene *Pch1* in wheat cultivars markedly reduces disease severity, as demonstrated by both visual assessments and qPCR analyses. Although minor decreases in fungicide sensitivity have been detected in *Oculimacula* populations, continued surveillance is essential to prevent the emergence of resistance. Therefore, integrated management strategies that combine the use of resistant cultivars, regular pathogen monitoring, and, when necessary, targeted fungicide applications are crucial for sustainable eyespot control and effective yield protection in wheat production.

## Figures and Tables

**Table 1:** Evaluation of eyespot symptoms on winter wheat cultivars after *Oculimacula* spp. inoculation (2022–2023).

Cultivar	*Pch1*	2022	2023	Mean
ANNIE	+	1.3	1.3	1.3
ILLUSION	+	2.3	1.2	1.7
PALLAS	+	1.8	1.7	1.8
KWS DONOVAN	+	2.0	1.7	1.9
CAMPESINO	+	1.9	2.0	2.0
LG ABSALON	+	2.4	2.3	2.4
ADINA	−	2.5	2.4	2.4
FAKIR	−	2.7	2.2	2.4
LG DITA	−	2.5	2.6	2.5
NONSTOP	−	2.7	2.6	2.6
LG MOCCA	−	2.5	2.9	2.7
GENIUS	−	2.6	3.0	2.8
BUTTERFLY	−	3.0	2.6	2.8
FRISKY	−	3.1	2.8	2.9
ASORY	−	2.5	3.5	3.0
BOHEMIA	−	2.9	3.1	3.0
LG MONDIAL	−	2.6	3.4	3.0
KALBEX	−	2.5	3.6	3.0
STEFFI	−	3.2	2.8	3.0
PIRUETA	−	2.9	3.1	3.0
CROSSWAY	−	2.5	3.6	3.1
SKIF	−	2.6	3.6	3.1
RGT SACRAMENTO	−	3.0	3.3	3.1
JULIE	−	3.3	3.0	3.1
DAGMAR	−	2.9	3.5	3.2
MERCEDES	−	3.5	3.2	3.3

Presence/absence of the *Pch1* gene (+/−). Scale 0–5 (0–2 resistant, 3 moderately resistant to moderately susceptible, 4–5 susceptible).

## Data Availability

The data can be provided by the authors.

## Supplementary Materials

The following supporting information can be downloaded at: https://www.mdpi.com/article/10.3390/plants15020285/s1. Table S1: Results of multiple pairwise comparisons (Z and adjusted *p* values) following the Kruskal—Wallis test for *Oculimacula* spp. sensitivity to fluxapyroxad between 2020 and 2025; Table S2: Results of multiple pairwise comparisons (Z and adjusted *p* values) following the Kruskal—Wallis test for *Oculimacula* spp. sensitivity to prothioconazole between 2020 and 2025; Figure S1: Eyespot symptoms on winter wheat; Figure S2: *Oculimacula yallundae* (4 weeks old culture) and *O. acuformis* (5 weeks old culture) on PDA.
