# Supplementary material for: Host Response of Winter Wheat to the Causal Agents of Eyespot and Fungicide Resistance of the Pathogens
Source: Plants (Basel). 2026 Jan 17;15(2):285. doi: 10.3390/plants15020285 (PMC12845032; doi:10.3390/plants15020285)

plants

MDPI

Article

Supplementary:

Table S1. Results of multiple pairwise comparisons (*Z* and adjusted *p* values) following the Kruskal–Wallis test for *Oculimacula* spp. sensitivity to fluxapyroxad between 2020 and 2025. The upper diagonal shows adjusted *p* values, and the lower diagonal lists the corresponding *Z* statistics. Although the Kruskal–Wallis test indicated a statistically significant overall difference between years ($H = 14.18$; $p = 0.0145$), no pairwise comparisons reached the significance threshold after adjustment.

Fluxapyroxad	2020	2021	2022	2023	2024	2025
2020		1.000	0.847	1.000	1.000	0.167
2021	0.328		0.316	0.977	1.000	0.060
2022	1.907	2.306		1.000	0.985	1.000
2023	1.456	1.844	0.463		1.000	1.000
2024	0.195	0.071	1.841	1.444		0.216
2025	2.538	2.880	0.879	1.289	2.448	

Table S2. Results of multiple pairwise comparisons (*Z* and adjusted *p* values) following the Kruskal–Wallis test for *Oculimacula* spp. sensitivity to prothioconazole between 2020 and 2025. The upper diagonal of the matrix shows *p* values adjusted for multiple testing, and the lower diagonal shows the corresponding Z statistics. Statistically significant differences ($p < 0.05$) are highlighted in red. The results indicate that the years 2021 and 2022 differed significantly from 2020 and 2023, confirming the temporal reduction in sensitivity observed in the boxplot analysis (Figure 2).

Prothioconazole	2020	2021	2022	2023	2024	2025
2020		<0.001	<0.001	1.000	0.270	0.063
2021	5.938		1.000	0.002	0.191	1.000
2022	5.681	0.572		0.002	0.100	0.986
2023	1.644	3.823	3.877		1.000	1.000
2024	2.366	2.490	2.713	0.846		1.000
2025	2.863	1.534	1.840	1.453	0.631	

Figure S1. Eyespot symptoms on winter wheat.

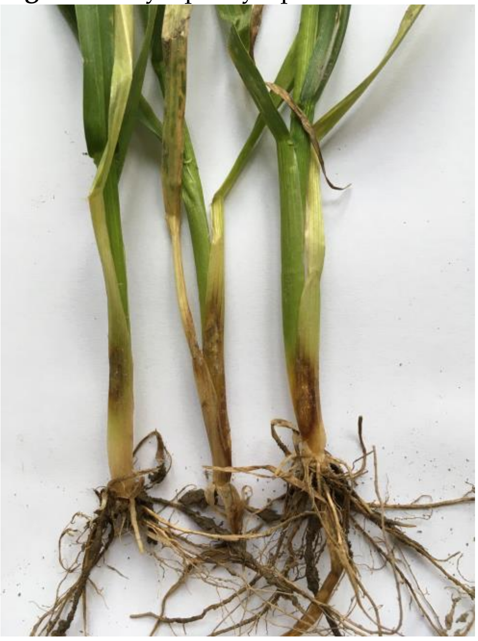

Figure S2. *Oculimacula yallundae* (4 weeks old culture) and *O. acuformis* (5 weeks old culture) on PDA.

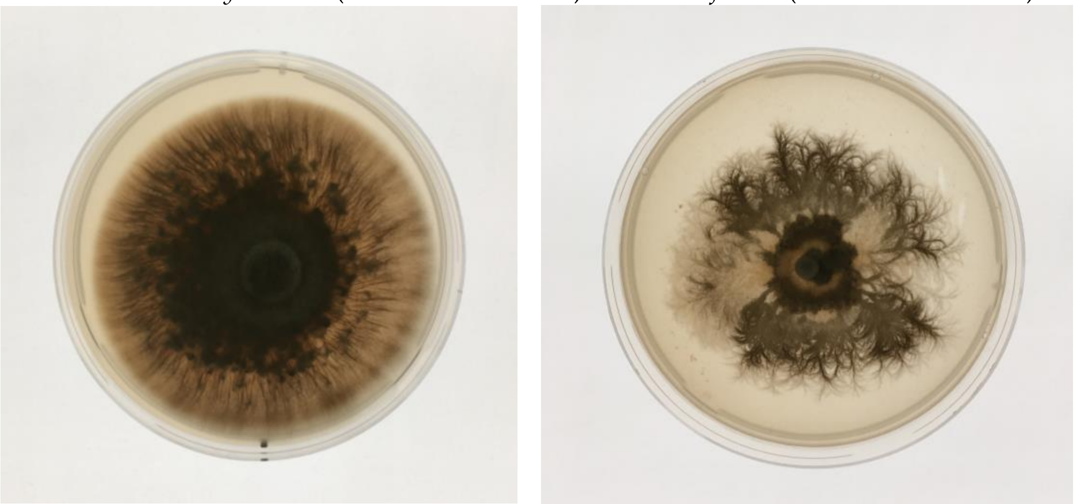

Supplement: Supplementary file 1 [file plants-15-00285-s001.zip › plants-4059275-supplementary.pdf]
